# Identification of Semiconductive Patches in Thermally Processed Monolayer Oxo‐Functionalized Graphene

**DOI:** 10.1002/anie.202004005

**Published:** 2020-05-27

**Authors:** Zhenping Wang, Qirong Yao, Christof Neumann, Felix Börrnert, Julian Renner, Ute Kaiser, Andrey Turchanin, Harold J. W. Zandvliet, Siegfried Eigler

**Affiliations:** ^1^ Freie Universität Berlin Institute for Chemistry and Biochemistry Takustraße 3 14195 Berlin Germany; ^2^ Physics of Interfaces and Nanomaterials University of Twente Enschede 7500 AE The Netherlands; ^3^ Friedrich Schiller University Jena Institute of Physical Chemistry Lessingstraße 10 07743 Jena Germany; ^4^ Universität Ulm Zentrale Einrichtung Elektronenmikroskopie Albert-Einstein-Allee 11 89081 Ulm Germany; ^5^ Current address: Max-Planck-Institut für Mikrostrukturphysik Weinberg 2 06120 Halle Germany

**Keywords:** electrical transport properties, graphene oxide, microscopy, oxo-functionalized graphene, semiconductors

## Abstract

The thermal decomposition of graphene oxide (GO) is a complex process at the atomic level and not fully understood. Here, a subclass of GO, oxo‐functionalized graphene (oxo‐G), was used to study its thermal disproportionation. We present the impact of annealing on the electronic properties of a monolayer oxo‐G flake and correlated the chemical composition and topography corrugation by two‐probe transport measurements, XPS, TEM, FTIR and STM. Surprisingly, we found that oxo‐G, processed at 300 °C, displays C−C sp^3^‐patches and possibly C−O−C bonds, next to graphene domains and holes. It is striking that those C−O−C/C−C sp^3^‐separated sp^2^‐patches a few nanometers in diameter possess semiconducting properties with a band gap of about 0.4 eV. We propose that sp^3^‐patches confine conjugated sp^2^‐C atoms, which leads to the local semiconductor properties. Accordingly, graphene with sp^3^‐C in double layer areas is a potential class of semiconductors and a potential target for future chemical modifications.

## Graphical abstract

**Localized semiconductive areas** are generated by thermal annealing of oxo‐functionalized graphene, by the formation of sp^3^‐patches isolating sp^2^‐graphene‐like structures. The semiconductive patches display a band gap of 0.4 eV. The discovery of the structural and electronic insights paves the way to novel semiconductors.

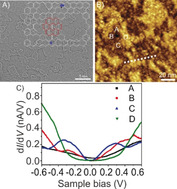

Graphene oxide (GO) is described as a derivative of graphene obtained by oxidation of graphite or graphene.^1^ The sp^2^‐bonded carbon atoms, which are arranged in a honeycomb lattice, are partially decorated with oxygen‐containing species.^2^ Tuning the sp^2^/sp^3^ ratio in the GO materials provides pathways to design diverse graphene derivatives with intriguing physicochemical properties including surface modifiability,^3^ tunable band gap,^4^ and variable luminescence^5^ for extensive applications in sensing based on electronic and luminescent devices.^6^ However, because of the polydisperse structure of GO, the structural model remains generalized, in particular with respect to the regiochemistry.2c, 7 During the preparation of GO via oxidation approaches such as Hummers’ method,^8^ over‐oxidation violently disintegrates the sp^2^‐carbon lattice and results in either vacancy defects on the scale of 10 nm at best or flake‐like amorphous carbon.^9^ The size of defect‐free graphene patches in reduced GO is about 1 nm.^10^ Over‐oxidation during the preparation of GO was identified as the reason for the ruptured graphene lattice in GO due to the loss of carbon via formation of CO_2_.^11^ As verified by Dimiev et al. using Hummers’ method in a first approximation, one CO_2_ molecule is formed from 20 carbon atoms.^12^ Recently, we found that kinetically controlled oxidation procedures can effectively hinder the over‐oxidation, and the oxidation can still be performed by harsh oxidants such as potassium permanganate in sulfuric acid or sodium chlorate in nitric acid.^13^ The obtained GO materials, which are a subclass of GO, are termed as oxo‐functionalized graphene (oxo‐G). The oxo‐G bears an intact carbon framework with densities of lattice defects of about 0.02 % and 0.5 %.^14^ It was demonstrated that hydroxyl, epoxy, and organosulfate groups decorate the carbon lattice on both sides of the basal plane and edge functional groups like carbonyl and carboxyl groups only play a minor role.^13^

The carbon lattice in oxo‐G can be visualized by high‐resolution transmission electron microscopy (TEM).^15^ Chemically processed oxo‐G with a degree of oxo‐functionalization of about 4 % (abbreviated as oxo‐G_4 %_) bears defect‐free areas with diameters of about 10 nm on average.^16^ After thermal processing up to 175 °C, the oxo‐G_4 %_ disproportionates and bears preserved graphene domains with diameters of about 3 nm, next to few‐atom large vacancy defects and holes with diameters of around 1–2 nm.^16^

Oxo‐G with a typical degree of functionalization of 60 % (oxo‐G_60 %_) displays a density of defects of about 2 % after annealing.^9^, ^14^ Those defects can act as structural motifs and active sites for selective chemical functionalization.^17^ So far, the vast majority of studies on GO or oxo‐G based materials mainly focused on optimizing preparation and reduction methods,^18^ understanding preparation protocols, probing the reduction mechanism,^19^ and developing applications.^20^ However, deep knowledge about the atomic structures and defects between oxidized and deoxygenated states in oxo‐G are ambiguous. In particular, it is still not clear how the structure of GO or oxo‐G_60 %_ evolves during thermal processing.

Here, we present the structure evolution and related transport properties of oxo‐G_60 %_ on the single‐layer level by gradual thermally induced disproportionation. The mobility values of monolayer oxo‐G first increased with the release of adsorbed water, disproportionation up to 220 °C and then decreased due to the formation of holes and surprisingly discovered stacked regions bearing sp^3^‐C. By X‐ray photoelectron spectroscopy (XPS), we identified a fraction of about 26 % C−C sp^3^ and about 3.4 % C−O/C−OH/C−O−C, containing nanometer‐sized sp^3^‐patches as visualized by TEM. Those regions turned out to be semiconducting with a band gap of 0.4 eV, as revealed by scanning tunneling spectroscopy (STS). Thus, sp^2^‐C isolated by sp^3^‐patches is most likely responsible for the local semiconducting behavior (see Scheme 1 and Figure 5).

**Scheme 1:**
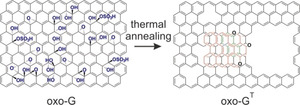
Schematic illustration of the chemical structure of oxo‐G and thermally processed oxo‐G (indicated as oxo‐G^T^). The latter results in the formation of holes and semiconducting sp^3^‐patches.

The starting oxo‐G material used here possesses a degree of functionalization of about 60 % sp^3^‐carbon, with hydroxyl, epoxy, and organosulfate groups as major functional groups.^21^

Temperature‐dependent electrical transport properties were studied by fabricating and analyzing a monolayer oxo‐G‐based field‐effect transistor (FET) device (Figure 1 A). The oxo‐G device was fabricated by deposition of a monolayer oxo‐G flakes on a heavily p‐doped Si substrate with a 300 nm thick SiO_2_ layer (Si/SiO_2_) using the Langmuir–Blodgett technique.^22^ Then, gold contacts were deposited on top of the monolayer oxo‐G flake by standard electron beam lithography (EBL) and gold evaporation. All electrical transport measurements were carried out with a two‐probe configuration (see Figure 1 B) under ambient conditions. The Si/SiO_2_ substrate serves as a back‐gate and gate dielectric. Different transport performances were obtained by iteratively heating the same device with the same oxo‐G flake from room temperature (RT) to 300 °C. All transfer characteristics (*I*_ds_‐*V*_bg_) reveal typical p‐type behavior (Figure 1 C–J). The large hysteresis between forward and reverse sweeps is induced by trapped charges.^23^ The resistance and charge carrier mobility are extracted from the transport curves in Figure 1 C–J. As depicted in Figure 1 K, on‐resistance of oxo‐G FET at *V*_ds_=0.5 V and *V*_bg_=0 V decreases from 5.3×10^8^ Ω to 3.3×10^5^ Ω. Evolution of the resistances reveals that the oxo‐G undergoes an insulator to conductor transition with a partial restoration of sp^2^‐carbon lattices in the oxo‐G flake by thermal processing. The change of hole mobilities (*μ*_h_) displays an inverted parabola shape. The *μ*_h_ of the untreated monolayer oxo‐G is 0.004 cm^2^ V^−1^ s^−1^ is very low, as expected due to the insulating nature. After thermal annealing up to 100 °C, the *μ*_h_ increases by an order of magnitude. This is because most polar adsorbents like water or oxygen molecules desorbed from the oxo‐G surface, as we identified before by thermogravimetric analysis.^11^ In addition, some decomposition of organosulfate groups takes place.^24^ But overall, the carbon skeleton of the oxo‐G remains relatively intact until 100 °C.^25^ Then, significant increase of the *μ*_h_ is observed between 140 °C and 220 °C. It can be deduced that the main deoxidation process occurs at this stage, which is accompanied by formation of π‐conjugated domains, in addition to vacancy defects, small holes, and CO_2_, as evidenced for oxo‐G_4 %_.^16^ The maximum *μ*_h_ of about 0.3 cm^2^ V^−1^ s^−1^ is obtained from oxo‐G^220°C^, which indicates the maximized sp^2^ graphene structures in oxo‐G^220°C^. In contrast, further annealing at higher temperature results in decreased *μ*_h_ values. These results clearly suggest the limited restoration of the graphene domains and irreversible structural decay of oxo‐G induced by the thermal processing.

**Figure 1:**
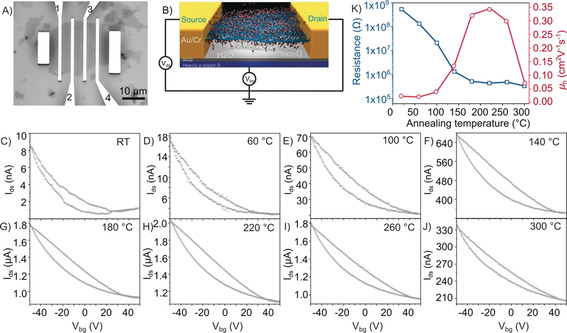
Electrical transport properties of a monolayer oxo‐G‐based FET device. A) An optical microscope image of a FET device with a monolayer oxo‐G flake as a channel. The distance between electrodes is 3 μm and the length of every electrode is 20 μm. B) Schematic view of the monolayer oxo‐G transistor with back‐gate two‐probe configurations. C–J) Room‐temperature transfer characteristics of monolayer oxo‐G treated by iteratively heating up to 300 °C. Metal contacts 1 and 2 were used as source and drain electrodes for the all measurements. K) Changes of resistance and mobility as a function of annealing temperature.

Next, XPS was conducted to analyze changes in the chemical composition of an iteratively annealed oxo‐G sample. The high‐resolution C 1s spectrum of oxo‐G in Figure 2 A displays a typical saddle‐like pattern, which stems from significant oxidation in oxo‐G. Four components assigned to C−C/C−H (51.8 %, at 284.6 eV), C−O/C−OH/C−O−C (40.6 %, at 286.7 eV), C=O (4.0 %, at 287.7 eV), and COOH (2.5 %, at 288.6 eV) are deconvoluted. The initial C/O ratio of oxo‐G was 2.2:1. No significant change of the chemical composition is detected up to 100 °C (Figure 2 B,C), in agreement with the results of transport measurements. However, starting at 140 °C, the intensity of the peak assigned to C−O bonds weakens significantly. A distinction between sp^2^‐ and sp^3^‐bonded C−C is observed and the sp^3^‐hybridized C−C bonds with 23.5 % are detected (Figure 2 D). The subsequent thermal treatments up to 300 °C do not induce an obvious change in chemical compositions (Figure 2 E–H), with the C−C sp^3^ reaching about 26.1 % and C−O/C−OH/C−O−C of 3.4 %. The corresponding C/O ratios increase slowly from 4.6 to 7.5 (Table S1). Thus, considering the relatively stable chemical composition but significantly weakened mobility values between 260 °C and 300 °C, we propose that structural rearrangements and formation of defects induced by thermal disproportionation further proceed.

**Figure 2:**
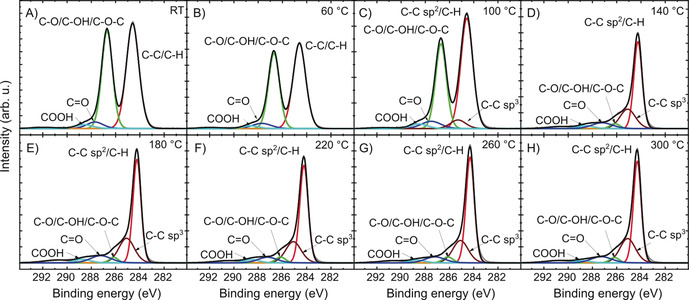
High‐resolution C 1s XPS of oxo‐G treated by iteratively annealing up to 300 °C.

However, the role of evolving C−C sp^3^‐carbon, as detected by XPS, remains unclear. To gain more precise structural insight into the thermally processed oxo‐G, TEM investigations were conducted.^16^, 21b, 26 The monolayer oxo‐G flakes were deposited onto a TEM sample grid, which subsequently was annealed at 300 °C in vacuum to induce the thermal disproportionation. While oxo‐G without thermal treatment possesses a relatively intact hexagonal carbon framework (Figure S2), monolayer oxo‐G^300°C^ shows an inhomogeneous structure as depicted in the chromatic (Cc) and spherical (Cs) aberration‐corrected high‐resolution TEM image presented in Figure 3. The hexagonal graphene structures are isolated by holes and stacked double‐layer patches, as marked. The size of the defect‐free graphene islands varies from 1 nm to 10 nm in diameter or length and these areas cover roughly 50 % of the whole surface. The observed holes with diameters of 3–5 nm comprise approximately 20 % of the area. In addition, the nanometer‐sized double‐layer regions distributed around holes are eye‐catching. Accordingly, sp^3^ arrangements in the stacked double‐layer regions are plausible in conjugation with sp^2^‐C.^27^

**Figure 3:**
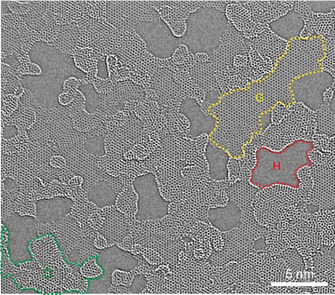
Cc/Cs‐corrected high‐resolution 80 kV TEM image of thermally processed oxo‐G at 300 °C (oxo‐G^300°C^), showing holes, areas of stacked carbon layers, and grain boundaries. The striking features are marked: holes (H), intact single‐layer graphene (G), and double‐layer carbon (D).

To further prove the presence and impact of the sp^3^‐areas, we conducted scanning tunneling microscopy (STM) and spectroscopy (STS). With STS we surprisingly found local semiconductor properties. First, the morphology of the oxo‐G^300°C^ was examined by STM. Figure 4 A shows a large‐scale STM image of a single oxo‐G^300°C^ flake on highly oriented pyrolytic graphite (HOPG). The average height of the single layer is about 2.0 nm, which is almost twice the thickness of monolayer oxo‐G, as we reported before.^28^ This is ascribed to fluctuations of the carbon plane caused by the rearrangement and loss of monoatomic carbon in oxo‐G after thermal annealing, as TEM showed. With increased magnification of the oxo‐G^300°C^ surface, dome‐shaped morphologies were detected (Figure 4 B). There are three differently colored distributions in Figure 4 B: dark, brown, and bright. Height profiles in Figure 4 C show the height difference from the bright plane to the brown plane, and from the dark plane to the brown plane of 0.9 and 1 nm, respectively. The topographical fluctuation over a 50 nm range is 1.9 nm, which nearly coincides with the thickness of this single layer. This indirectly indicates that the dark, brown, and bright regions correspond to holes, graphene domains, and stacked double‐layer carbon, respectively.

**Figure 4:**
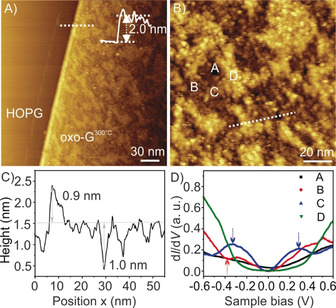
A) Large‐scale STM topographic image of oxo‐G^300°C^ on HOPG (200 nm × 200 nm; tunneling current *I*_t_=0.5 nA, sample voltage bias *V*_s_=−0.6 V). The inset is the height profile of the monolayer oxo‐G^300°C^ flake on HOPG. B) STM topographic image obtained at higher magnification of the surface of the oxo‐G^300°C^ flake shown in (A) (100 nm × 100 nm; *I*_t_=0.5 nA, *V*_s_=−0.3 V). C) Height profiles along the dashed line marked in (B). D) Local d*I*/d*V* curves measured at positions marked in (B).

The local electronic properties of these heterogeneous topographical surfaces in the oxo‐G^300°C^ sample were investigated via STS. The differential conductivity (d*I*/d*V*), which is proportional to the local density of states (LDOS) at small bias, was simultaneously obtained during the STM measurements using a grid *I–V* scan. The d*I*/d*V* curves in Figure S4 were obtained by averaging 3600 d*I*/d*V* curves recorded on the HOPG and the oxo‐G^300°C^ surface at respective places, respectively, as labeled in Figure 4 B. The d*I*/d*V* spectrum of HOPG shows a nearly symmetrical parabolic geometry. The oxo‐G^300°C^ exhibits a V‐shaped d*I*/d*V* reminiscent of two‐dimensional Dirac material. The Dirac point is located at +40 mV. This p‐type doping here is in agreement with the transport measurements in Figure 2 J determined on micrometer‐sized channels. The specific electronic information at different positions (marked as A, B, C, and D, shown in Figure 4 B) was depicted by the local d*I*/d*V* spectra in Figure 4 D (individual data shown in Figure S8). Obviously, the measured four positions present a distinct electrical inhomogeneity. First, the black line (measured at dark areas such as position A) shows a metallic‐like behavior, similar to the LDOS behavior of HOPG,^29^ which confirms that the dark areas are holes. Then, the red line (measured at brown areas like position B) shows a conical‐shaped curve, corresponding to single‐layer graphene structures.^30^ It is worth noting that the fluctuations marked with the red arrow represent defective states, indicating some defects exist in the single‐layer graphene structure. Two prominent peaks marked with blue arrows are observed in the blue line (measured at bright areas like position C). Similar STS spectra were also found in twisted graphene bilayers.^31^ The two saddle peaks are attributed to energy separations of the low‐energy van Hove singularities (VHSs) in graphene bilayers. Therefore, it can be demonstrated that the bright regions contain some sp^2^‐hybrided double‐layer graphene structures. It is in particular interesting that a suppressed d*I*/d*V* distribution (green line) is measured at the brighter areas (position D, cf. Figure S8). The green averaged d*I*/d*V* curve (Figure 4 D) represents typical semiconducting behavior15b, 32 with a band gap of around 0.4 eV (Figure 4 D). Combining the atomically resolved carbon structures (Figure 3 and Figure 4 B) with the height of 1.9 nm (Figure 4 C) at position D, we thereby deduce that such a large band gap can be attributed to formed conjugated sp^2^‐C, which is isolated from the surrounding graphene lattice.

As detected by XPS, the sp^3^‐sp^3^‐C and C−O/C−OH/C−O−C of 3.4 %. can act as insulators (illustrated in Figure 5). Their appearance might be related to the in‐plane disruption of carbon–carbon bonds during the formation of holes, whereby the released carbon fragments react with the underlying graphene by sp^3^‐hybridization (Figure 5). Since the semiconducting areas make up 25 % (based on TEM area analysis) of the entire carbon layer, their effect on the overall band structure of oxo‐G^300°C^ is almost insignificant. The formation of sp^2^ carbon upon thermal disproportionation of oxo‐G is supported by FTIR investigations in Figure S5, due to the IR‐active signal at about 1570 cm^−1^, a signal that was also found in nanodiamonds with sp^2^‐patches.^33^ Moreover C−H bond cleavage may play a role, as evidenced by FTIR (2920 cm^−1^ and 2850 cm^−1^, Figure S5); however, elimination of water is more likely up to 140 °C.

**Figure 5:**
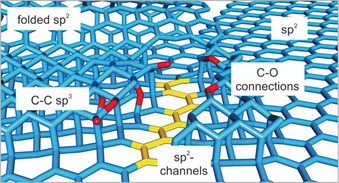
Schematic illustration of a proposed chemical structure of thermally processed oxo‐G, accounting for mixed sp^2^‐ and sp^3^‐C structures, which include ether‐like connections and possibly carbonyl and hydroxyl groups at the rims. The idealized sp^3^‐structures insulate conjugated sp^2^‐channels, which are identified as semiconductive.

In summary, it can be stated that sp^3^‐sp^3^ diamond‐like, imperfect sp^3^‐sp^2^‐sp^3^, and C−O−C bridged out‐of‐plane structures open a new path to semiconducting graphene‐based materials. Here, we describe the defect structures including holes and bilayer sp^3^‐patches induced by thermal disproportion of the oxo‐G. The identified C−C sp^3^‐patches and bridging C−O−C motifs, which are connected to nm‐sized patches of the hexagonal carbon lattice of graphene, are separated by grain boundaries and holes that are 5 nm in diameter. We suggest that C−C sp^3^‐bonds are formed either after folding or adsorption of carbon patches, indicating that reactive species, including C−O structures, are formed in the course of the disproportionation reaction. The sp^3^‐patches isolate residual sp^2^‐C and thus local STS reveals the semiconducting behavior of these areas. It turns out that the nm‐sized mixed sp^2^‐ and sp^3^‐structures have a band gap of ≈0.4 eV. Our study indicates that semiconductor/graphene hybrid materials are interesting materials with local semiconducting properties. With this deeper insight into the thermal disproportionation of oxo‐G and correlation to the electrical properties, future applications and the development of carbon‐based semiconductors becomes possible. In particular, the formation of holes and sp^3^‐stacked regions potentially plays a significant role for chemical reactions used to post‐functionalize materials. Moreover, bottom‐up synthesized molecular carbon materials containing sp^3^‐ and sp^2^‐carbon with a tunable band‐gap might be discovered in the future.

## Conflict of interest

The authors declare no conflict of interest.

## Supporting information

As a service to our authors and readers, this journal provides supporting information supplied by the authors. Such materials are peer reviewed and may be re‐organized for online delivery, but are not copy‐edited or typeset. Technical support issues arising from supporting information (other than missing files) should be addressed to the authors.

Supplementary
