# Supplementary material for: Identification of Semiconductive Patches in Thermally Processed Monolayer Oxo‐Functionalized Graphene
Source: Angew Chem Int Ed Engl. 2020 May 27;59(32):13657–62. doi: 10.1002/anie.202004005 (PMC7496721; doi:10.1002/anie.202004005)
Supplement: Supplementary file 1 — Supplementary [file ANIE-59-13657-s001.pdf]

Angewandte Chemie
Eine Zeitschrift der Gesellschaft Deutscher Chemiker

Supporting Information

Identification of Semiconductive Patches in Thermally Processed Monolayer Oxo-Functionalized Graphene

*Zhenping Wang, Qirong Yao, Christof Neumann, Felix Börrnert, Julian Renner, Ute Kaiser, Andrey Turchanin, Harold J. W. Zandvliet, and Siegfried Eigler**

anie_202004005_sm_miscellaneous_information.pdf

SUPPORTING INFORMATION

Table of Contents

EXPERIMENTAL PROCEDURES 2
MATERIALS: 2
SYNTHESIS OF OXO-G: 2
PREPARATION OF LB-ASSEMBLED OXO-G FILM: 2
FABRICATION OF MONOLAYER OXO-G FET TRANSISTOR: 2
Preparation of GO Films on ZnSe 2
CHARACTERIZATION: 2
FIGURE S1 3
FIGURE S2 3
FIGURE S3 4
FIGURE S4 4
FIGURE S5 5
FIGURE S6 5
FIGURE S7 6
TABLE S1 6
FIGURE S8 8

Experimental Procedures

Materials:

Double-distilled water was obtained from Carl Roth GmbH. Graphite (3061) was purchased from Asbury Carbon. Chemicals, solvents and other materials were obtained from Sigma-Aldrich. Si wafers with a 300 nm thick SiO_2 layer were purchased from Fraunhofer-Institut für Integrierte Systeme und Bauelementetechnologie IISB in Erlangen.

Synthesis of oxo-G:

Oxo-G was synthesized by low-temperature oxidation of graphite based on our previously reported method. Briefly, 1g of graphite (type 3061, Asbury Carbon) was mixed with 25 mL of sulfuric acid (97.5%) at a temperature low than 10 °C. Then, 2 g of $KMnO_4$ was slowly added over 4 h and further stirred for 16 h. After that, 20 mL of cold diluted sulfuric acid (20 wt%) and 50 mL of cold double distilled water were orderly added into the mixture using programmed pump with 4 h and 16 h. Subsequently, 20 mL of H_2O_2 (5 wt%) was added to remove excess manganese species. Finally, the oxo-G dispersion solution was obtained after purification treatment by centrifugation and mild sonication.

Preparation of LB-assembled oxo-G film:

The oxo-G spreading solution (2 mL) with a 1:2 mixture of water/ methanol was added on a water subphase trough (Kibron, µtrough). Flakes of oxo-G were deposited onto Si/SiO_2 (300 nm) substrate (for preparation of XPS samples and FET devices), HOPG bulk (for preparation of STM samples) and Quantifoil grid (for preparation of TEM samples) at 3 mN/m by Langmuir–Blodgett (LB) technique.[21] The LB-assembled oxo-G samples were used for further characterization and preparation of FET devices.

Fabrication of monolayer oxo-G FET transistor:

Standard electron beam lithography (EBL) procedure (Raith PIONEER TWO) was used to define and expose the geometry of metal contacts. Subsequently, a 5 nm/ 70 nm Cr/Au stack was deposited with thermal evaporation (Kurt J. Lesker NANO 36) and lifted off in acetone to make electrode contact to oxo-G flake. Thermally annealing of the oxo-G FET device was performed using a heating oven with a vacuum degree of 10^{-3} mbar.

Preparation of GO Films on ZnSe

A diluted oxo-G solution was drop-casted on a ZnSe substrate. And then the oxo-G film was formed by evaporation of the distilled water at ambient conditions.

Characterization:

Optical microscope (Nikon Eclipse, LV150) was used to visualize oxo-G FET devices. Cc/Cs-corrected high-resolution 80 kV Transmission electron microscopy (TEM) images were obtained using the SALVE microscope operated at 80 kV electron acceleration voltage. Statistical Raman spectroscopy (SRS) was recorded using a Horiba Explorer spectrometer with a 532 nm laser for excitation. Scanning tunneling microscopy (STM) and Scanning tunneling spectroscopy (STS) were achieved by Omicron-STM1 with an ultra-high vacuum of <10^{-10} mbar. X-ray photoelectron spectroscopy (XPS) was performed by a multiprobe system (Scienta Omicron) with a monochromatic X-ray source (Al K_α) and an electron analyzer (Argus CU) with 0.6 eV spectral energy resolution. The spectra were fitted using Voigt functions (30: 70) after Shirley background subtraction. The FTIR measurements were performed by FT-IR Vertex 70 (Bruker) in transmission mode using ZnSe windows as support. All transport measurements were performed at ambient conditions by a two-probe station and two source-measurement units (Keithley 2450).

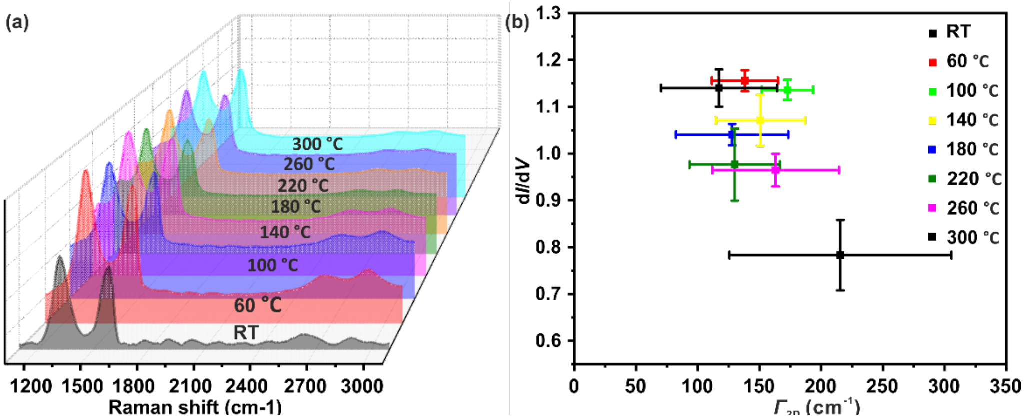

Figure S1. Statistical Raman analysis performed by scanning an area of with ~ 20 ×20 μm^2 at 532 nm laser excitation wavelength. (a). Average Raman spectra of monolayer oxo-G and thermally processed monolayer oxo-G at different temperature. (b) plot of I_D/I_G versus Γ_{2D}.

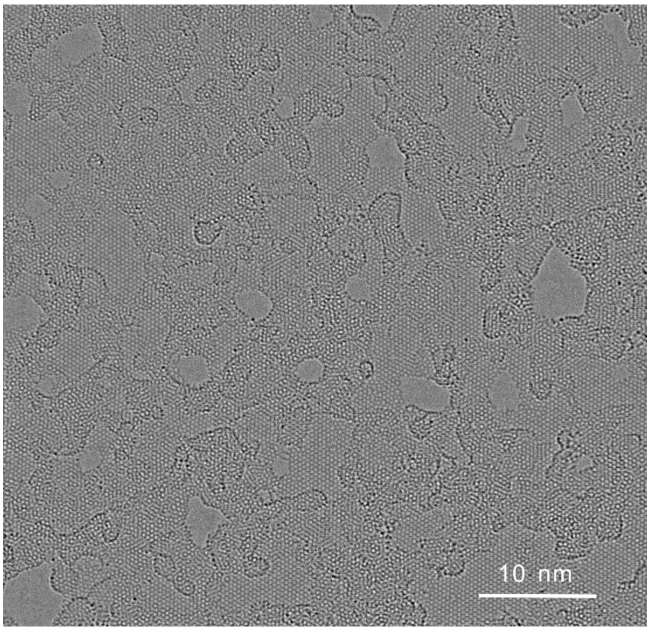

Figure S2. Cc/Cs-corrected high-resolution 80 kV Transmission electron microscopy (TEM) images of untreated oxo-G.

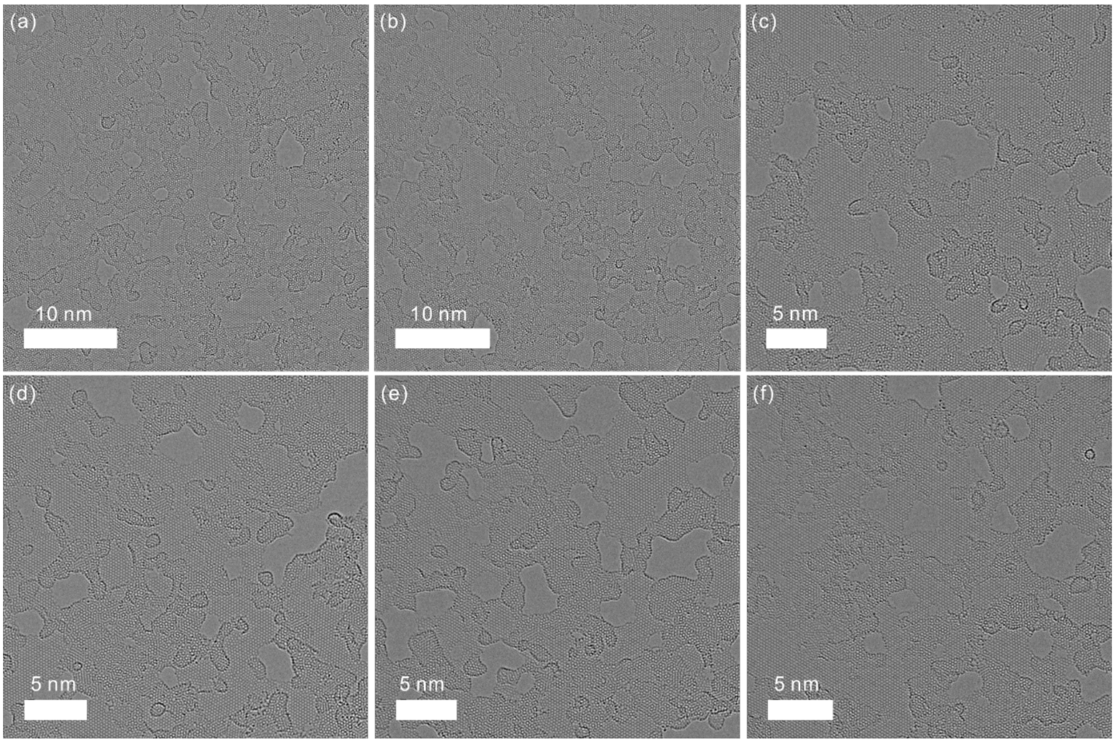

Figure S3. Cc/Cs-corrected high-resolution 80 kV Transmission electron microscopy (TEM) images of oxo-G treated at 300 °C for 15 mins. The oxo-G sample was heated using the TEM heating holder in the vacuum of our plasma cleaner at 300 °C for 15 minutes and analyzed directly afterwards in the SALVE microscope at 80 kV electron acceleration voltage.

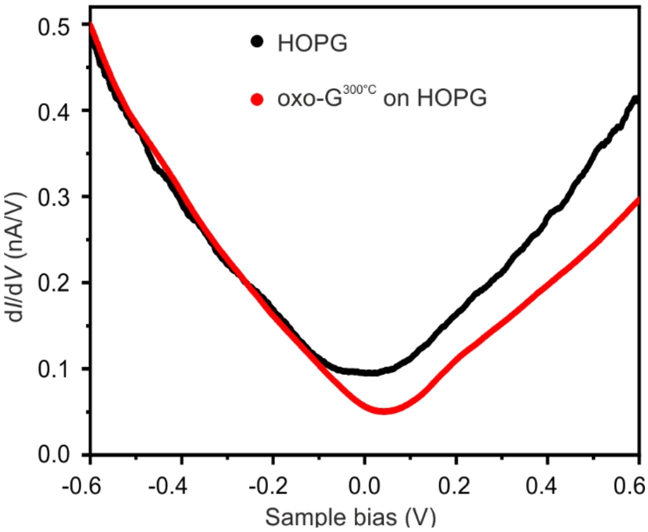

Figure S4. Averaged d*I*/d*V* spectra on HOPG and oxo-G$^{300 °C}$ surface. The d*I*/d*V* spectra were recorded by using a lock-in amplifier with a sinusoidal modulation (*f* =1.89 KHz, V_{mode} = 18 meV) of the feedback-loop gain off.

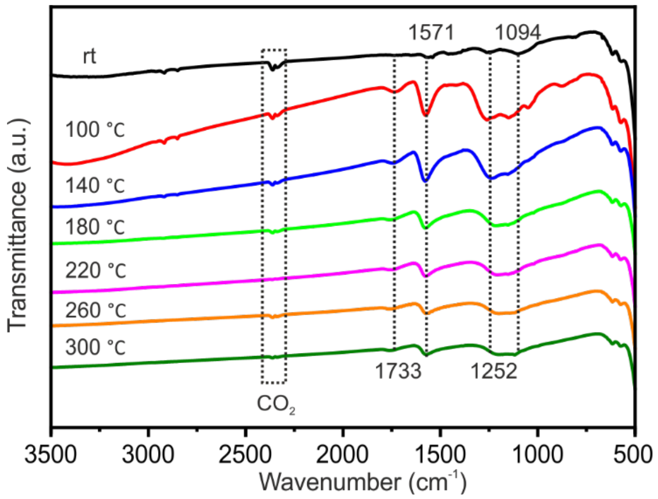

Figure S5. FTIR spectra of thermally processed oxo-G films on a ZnSe substrate.

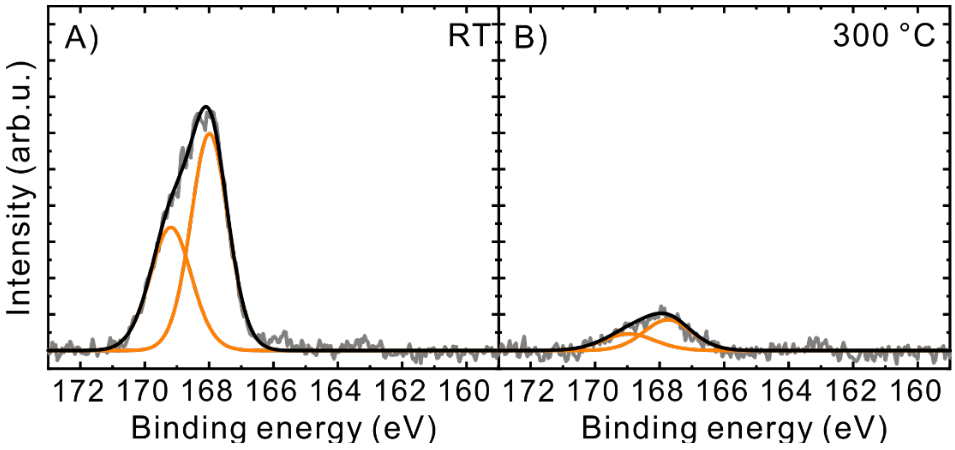

Figure S6. High-resolution S 2p XPS of oxo-G and oxo-G^T annealed with 300 °C. Both spectra show a signal assigned to oxidized sulfur whereas no trace of C-S bonds (162-164 eV) are detected. The total amount of sulfur is decreasing with higher annealing temperatures (Table S1).

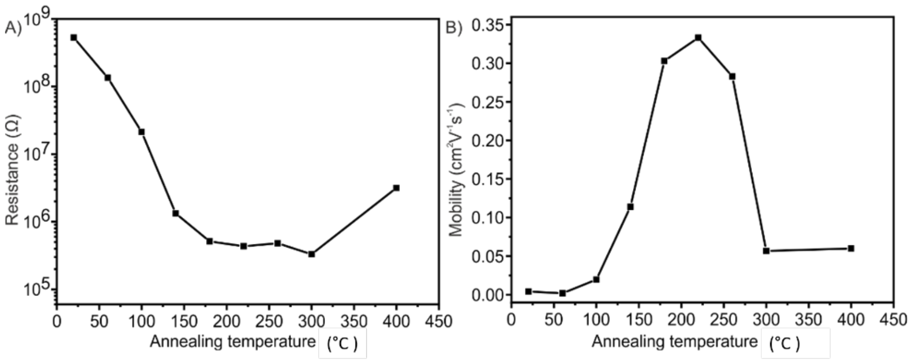

Figure S7. Changes of resistance and mobility as a function of annealed temperature.

Table S1. C/O and C/S ratios obtained by X-ray photoelectron spectroscopy using a relative sensitivity factor (RSF) of 1 (C 1s), 2.93 (O 1s) and 1.68 (S 2p). Note that the absolute C/O ratio is affected by the oxygen in the underlying SiO_2/Si substrate.

sample	C / O ratio (±0.1)	C / S ratio (±1)
RT	2.2 : 1	24 : 1
60 °C	2.4 : 1	28 : 1
100 °C	2.4 : 1	28 : 1
140 °C	4.6 : 1	32 : 1
180 °C	4.7 : 1	33 : 1
220 °C	4.6 : 1	33 : 1
260 °C	5.0 : 1	79 : 1
300 °C	7.5 : 1	191 : 1

Table S2. Quantitative analysis of the high resolution XP C 1s spectra presented in **Figure 1** of the main paper including the peak assignment, their binding energies, full width at half maximum (FWHM) values and areas obtained from the spectra deconvolution.

Peak assignment	Binding energy, eV	FWHM, eV	Area, %
**RT**			
C-C/C-H	284.6	1.2	51.8
C-O/C-OH/C-O-C	286.7	1.0	40.6
C=O	287.7	1.6	4.0
COOH	288.6	1.6	2.5
aromatic shake-up	291.8	2.5	1.1

**60 °C**			
C-C/C-H	284.6	1.2	52.3
C-O/C-OH/C-O-C	286.7	1.1	39.5
C=O	287.7	1.8	4.7
COOH	288.6	1.5	2.1
aromatic shake-up	291.9	2.5	1.4
**100 °C**			
C-C sp^2/C-H	284.6	1.2	50.7
C-C sp^3	285.3	1.5	5.1
C-O/C-OH/C-O-C	286.7	1.0	34.9
C=O	287.5	1.9	5.4
COOH	288.5	1.6	2.9
aromatic shake-up	291.5	2.5	1.0
**140 °C**			
C-C sp^2/C-H	284.3	0.8	55.6
C-C sp^3	285.1	1.5	23.5
C-O/C-OH/C-O-C	286.1	0.9	3.7
C=O	287.2	2.0	9.3
COOH	288.6	1.5	3.5
aromatic shake-up	290.6	2.5	4.4
**180 °C**			
C-C sp^2/C-H	284.2	0.8	54.2
C-C sp^3	285.1	1.5	24.7
C-O/C-OH/C-O-C	286.2	0.8	3.1
C=O	287.2	2.0	9.6
COOH	288.7	1.5	3.6
aromatic shake-up	290.8	2.5	4.9
**220 °C**			
C-C sp^2/C-H	284.2	0.8	57.5
C-C sp^3	285.1	1.5	23.2
C-O/C-OH/C-O-C	286.2	0.9	3.9
C=O	287.2	1.9	7.7
COOH	288.5	1.5	3.5
aromatic shake-up	290.5	2.5	4.3
**260 °C**			
C-C sp^2/C-H	284.3	0.8	58.2
C-C sp^3	285.1	1.5	23.1
C-O/C-OH/C-O-C	286.1	1.0	4.0
C=O	287.2	1.9	7.9
COOH	288.7	1.5	2.9
aromatic shake-up	290.7	2.5	4.0

**300 °C**			
C-C sp^2/C-H	284.3	0.8	54.9
C-C sp^3	285.1	1.5	26.1
C-O/C-OH/C-O-C	286.2	0.9	3.4
C=O	287.3	2.0	8.9
COOH	288.9	1.5	2.4
aromatic shake-up	290.8	2.5	4.4

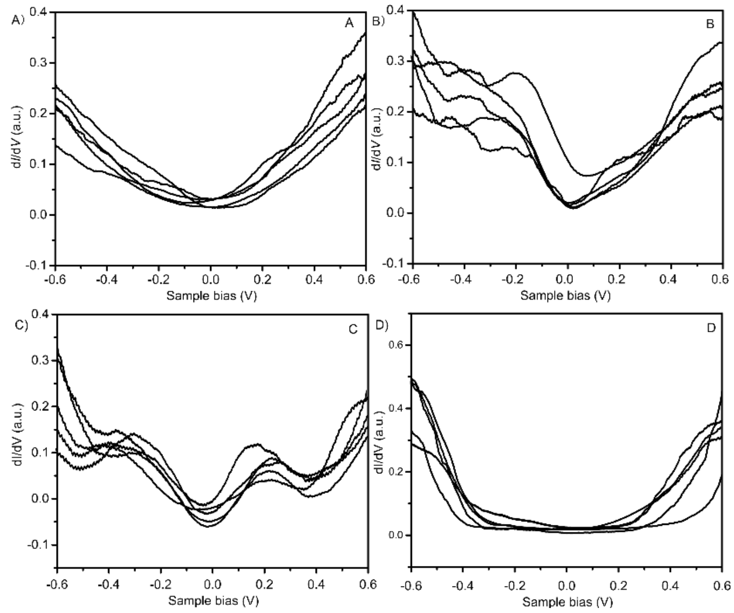

Figure S8. STS spectra of the holes (A), graphene domains (B) and stacked bilayer patches (C) And (D). The five curves in each figure were captured from five different domains in one sample.
